# The repertoire of yeast species from human clinical samples in the United Kingdom 2016–2022: epidemiology, clinical relevance, and fluconazole resistance

**DOI:** 10.1128/spectrum.01360-26

**Published:** 2026-07-06

**Authors:** Michael J. Wilson, Sue McLachlan, Ciara Mann, Martin Gough, Phillipa Brown, Cheryl Yung, Michael D. Palmer, Alireza Abdolrasouli, Elizabeth M. Johnson, Andrew M. Borman

**Affiliations:** 1 UK National Mycology Reference Laboratory, UK Health Security Agency South-West, Southmead Hospital 436286 , Bristol, United Kingdom; 2 North Bristol NHS Trust, Southmead Hospital 1982, Bristol, United Kingdom; 3 MRC Centre for Medical Mycology, University of Exeter 3286, Exeter, United Kingdom; Mayo Foundation for Medical Education and Research, Rochester, Minnesota, USA

**Keywords:** yeast, epidemiology, United Kingdom, clinical relevance, antifungal resistance

## Abstract

The United Kingdom National Mycology Reference Laboratory (MRL) performs routine identification and susceptibility testing of isolates of pathogenic yeasts from diverse clinical samples submitted from across the UK. Here, we extracted data from our laboratory information management system for all yeasts referred to the laboratory for the period 2016–2022. Data for a total of 17,631 isolates comprising 95 species were retrieved and analyzed according to frequency of recovery from each clinical sample type and anatomical site. In agreement with most international epidemiological surveys, *Candida albicans* and *Nakaseomyces glabratus* (syn. *Candida glabrata*) were the two most common yeast species identified, comprising cumulatively over 66% of all isolates referred to the MRL. *C. albicans* and *N. glabratus* predominated in frequency from all anatomical sites except the central nervous system, where *Cryptococcus neoformans* was the most frequently encountered, and respiratory secretions from cystic fibrosis patients and ocular samples, where *Candida parapsilosis* prevailed. The diversity of different species recovered from sputum, and the oral cavity was predictably large, in keeping with the contamination of the oropharynx by myriad yeasts introduced with food and beverages, in addition to those organisms that naturally occur as commensals on upper respiratory surfaces. More surprising was the diversity of organisms recovered from blood cultures, with 60 different species encountered during the study period. Finally, analysis of fluconazole susceptibility data for *C. albicans*, *C. parapsilosis*, and *C. tropicalis* demonstrated that fluconazole resistance in these organisms is currently reassuringly rare in the UK except in isolates causing superficial mucosal infections.

**IMPORTANCE:**

Here, we analysed over 17,000 isolates of yeast by anatomical/clinical site, highlighting an enormous range of species (>60) that can cause fungemia. Our data highlight that resistance to fluconazole in key pathogenic *Candida* species (*C. albicans, C. tropicalis, C. parapsilosis*) is currently rare in isolates from deep sites in the United Kingdom.

## INTRODUCTION

The unicellular *fungi* loosely described as yeasts encompass in excess of 2,000 known species distributed across the *Ascomycota* and *Basidiomycota*, with conservative estimates suggesting that the true number of species might be 100-fold higher (1, 2). Yeasts are ubiquitous in nature (3), essential constituents of many foods and beverages (3, 4), and also widespread in wild and domesticated animals and humans, either as commensal organisms or pathogens depending on the immune status of the host and the site of colonization or overgrowth (3, 5–10). Invasive infections caused by *Candida* species and allied yeast genera are the leading cause of fungal deaths worldwide, with *Candida albicans* and *Nakaseomyces glabratus* (syn. *Candida glabrata*) predominating in most geographic areas (9, 11–14). While isolation of a yeast from a normally sterile site, together with appropriate symptoms, is usually diagnostic of invasive or disseminated disease, the ubiquitous nature of most yeast species renders isolation of organisms from non-sterile sites more difficult to interpret and less than proof of clinical significance (15–17).

The increasing number of patients at risk of invasive fungal infections, coupled with improvements in identification methods, has driven a dramatic increase in the spectrum of yeast isolates identified from clinical samples (11–14, 18–21). It is widely accepted that different yeast species have species-specific antifungal susceptibility profiles, and species-specific interpretive breakpoints have been introduced by EUCAST and CLSI (22–24). Thus, accurate identification of isolates to the species level is paramount for optimal therapeutic decisions and patient management. The UK Health Security Agency National Mycology Reference Laboratory (MRL, Bristol, United Kingdom) receives large numbers of isolates of diverse yeast species from a wide range of clinical samples for identification and/or susceptibility testing each year. We have previously published surveys for resistance rates to fluconazole and other systemic antifungal agents for the yeast species more commonly encountered in our laboratory (12, 21). However, those studies did not address the question of whether certain species were associated with specific anatomical sites or whether resistance rates for common species with fluconazole varied by isolation site. Here, we present an analysis of ~17,000 yeast isolates referred to the MRL during the period 2016–2022 triaged by the most common clinical sample type and anatomical site (excluding isolates with unknown/not stated sites of isolation). Species-specific associations with different anatomical sites and fluconazole resistance rates in *C. albicans*, *C. tropicalis*, *C. parapsilosis*, and *N. glabratus* are presented.

## MATERIALS AND METHODS

### Clinical isolates

Isolate identification, site of isolation, and antifungal susceptibility data were collected from our laboratory information management system (LIMS) for all clinical yeast isolates referred to the MRL between 2016 and 2022 and categorized by site of isolation and sample type. Isolates from skin swabs collected for screening purposes (for example, to detect *Candidozyma auris*) were not included within the LIMS search as their coding data did not contain the requisite information on the specific sample site.

Between 2016 and 2022, 17,631 isolates of yeasts (approximately 2,500 isolates per year) were submitted to the MRL for identification and/or susceptibility testing from the following stated anatomical sites: genital (*N* = 5,802 isolates), of which 1,916 were denoted as genital-recurrent infection; blood (*N* = 4,538); sputum (*N* = 2,195), of which 156 were identified as from patients with cystic fibrosis (CF); oral (*N* = 1,955); urine (*N* = 1,802); bronchoalveolar lavage (BAL) fluid (*N* = 690); eyes, including contact lenses (*N* = 254); speech box or voice prosthesis (*N* = 148); central nervous system (CNS) including cerebrospinal fluid (CSF; *N* = 141); and pleural fluid (*N* = 106; Table 1). A further ~2,500 isolates across the entire study period (~15% of all isolates received) were excluded from the analysis as no anatomical site of isolation was specified. Isolates included examples of common and rarer *Candida* species, allied ascomycetous yeast species subsequently reclassified in alternative genera (25), and also a number of basidiomycetous yeasts.

**TABLE 1:** Number of isolates received from each sample site and corresponding number of species identified^*a*^

Site	Isolates received (% of total)	Species identified
Blood culture	4,538 (25.7%)	60
Genital	3,886 (22.0%)	37
Sputum	2,039 (11.6%)	54
Oral	1,955 (11.1%)	48
Genital-recurrent	1,916 (10.9%)	19
Urine	1,802 (10.2%)	29
BAL fluid	690 (3.9%)	37
Eyes/contact lens	254 (1.4%)	22
CF sputum	156 (0.9%)	29
Speech box	148 (0.8%)	17
CNS	141 (0.8%)	11
Pleural fluid	106 (0.6%)	16
Total	17,631	95

^*a*^ BAL, bronchoalveolar lavage; CF, cystic fibrosis; CNS, central nervous system.

### Yeast identification and antifungal susceptibility testing

All isolates were identified by matrix-assisted laser desorption/ionization time-of-flight mass spectrometry (MALDI-TOF MS) using the Bruker Biotyper Sirius Platform as previously described (18), with 26S rRNA gene amplification and sequencing (primers LSUFP and LSURP, [19]) used in cases of identification failure. Antifungal drug susceptibility testing was performed according to CLSI guidelines by broth microdilution, exactly as described previously (26), with MICs interpreted using established CLSI breakpoints (22).

## RESULTS

Overall, 95 different species were identified from the 17,631 clinical isolates submitted to the MRL between 2016 and 2022 (Table 2). Of the species we identified in this study, 66/95 (69.5%) were *Ascomycota*, and 18/95 (18.9%) were *Basidiomycota*. The remaining 1/95 (1.1%) was *Prototheca* spp., and while not a true fungus, it is included here given the morphological similarity with yeasts.

**TABLE 2:** Total list and number of each species identified (previous organism names in parentheses)^*a*^

Organism	Total	%
*Candida albicans*	7,381	41.86
*Nakaseomyces glabratus* (*Candida glabrata*)	4,279	24.27
*Candida parapsilosis*	1,266	7.18
*Candida dubliniensis*	903	5.12
*Candida tropicalis*	851	4.83
*Saccharomyces cerevisiae*	479	2.72
*Clavispora lusitaniae* (*Candida lusitaniae*)	451	2.56
*Pichia kudriavzevii* (*Candida kruzei*)	440	2.5
*Meyerozyma guilliermondii* (*Candida guillermonidii*)	242	1.37
*Cryptococcus neoformans*	204	1.16
*Rhodotorula mucilaginosa*	112	0.64
*Kluyveromces marxianus* (*Candida kefyr*)	108	0.61
*Meyerozyma caribbica* (*Candida fermentati*)	101	0.57
*Candidozyma auris* (*Candida auris*)	89	0.5
*Candida metapsilosis*	72	0.41
*Magnusiomyces capitatus* (*Geotrichum capitatum*)	63	0.36
*Candida orthopsilosis*	43	0.24
*Cyberlindnera fabianii* (*Candida fabianii*)	38	0.22
*Geotrichum candidum*	33	0.19
*Naganishia diffluens* (*Cryptococcus diffluens*)	33	0.19
*Trichosporon asahii*	33	0.19
*Wickerhamomyces anomalus* (*Candida pelliculosa*)	34	0.19
*Pichia cactophila* (*Candida inconspicua*)	25	0.14
*Lodderomyces elongisporus*	21	0.12
*Nakaseomyces nivariensis* (*Candida nivariensis*)	20	0.11
*Yarrowia lipolytica* (*Candida lipolytica*)	20	0.11
*Apiotrichum mycotoxinivorans* (*Trichosporon mycotoxinovorans*)	17	0.1
*Wickerhamiella pararugosa* (*Candida pararugosa*)	16	0.09
*Malassezia furfur*	15	0.09
*Malassezia pachydermatis*	13	0.07
*Rhodotorula dairenensis*	13	0.07
*Candidozyma haemuli* (*Candida haemulonii*)	12	0.07
*Tardiomyces blankii* (*Candida blankii*)	12	0.07
*Trichosporon inkin*	12	0.07
*Prototheca* spp.	11	0.06
*Pichia kluyveri*	10	0.06
*Pichia fermentans* (*Candida lambica*)	9	0.05
*Pichia barkerii*	8	0.05
*Wickerhamomyces onychis*	8	0.05
*Hanseniospora guilliermondii*	7	0.04
*Pichia manshurica*	7	0.04
*Candida palmioleophila*	6	0.03
*Debaryomyces hansenii* (*Candida famata*)	6	0.03
*Kodamaea ohmeri* (*Candida guilliermondii* var. membranifaciens)	6	0.03
*Cyberlindnera jadinii* (*Candida utilis*)	5	0.03
*Metschnikowia pulcherrima* (*Candida pulcherrima*)	5	0.03
*Pichia norvegensis* (*Candida norvegensis*)	5	0.03
*Sporopachydermia cereana*	5	0.03
*Sungouiella intermedia* (*Candida intermedia*)	4	0.02
*Starmerella magnoliae*	4	0.02
*Starmerella sorbosivorans*	4	0.02
*Trichosporon japonicum*	4	0.02
*Blastobotrys adeninivorans*	3	0.02
*Candida zeylanoides*	3	0.02
*Cutaneotrichosporon cutaneum* (*Trichosporon cutaneum*)	3	0.02
*Cutaneotrichosporon mucoides* (*Trichosporon mucoides*)	3	0.02
*Diutina catenulata*	3	0.02
*Geotrichum silvicola*	3	0.02
*Kazachstania telluris*	3	0.02
*Pichia occidentalis*	3	0.02
*Arthrographis kalrae*	2	0.01
*Pichia ethanolica* (*Candida ethanolica*)	2	0.01
*Candida sanyaensis*	2	0.01
*Hansensiospora uvarum*	2	0.01
*Millerozyma farinosa*	2	0.01
*Nakaseomyces bracarensis* (*Candida bracarensis*)	2	0.01
*Starmerella stellata*	2	0.01
Total = 17,631		

^*a*^ Plus 1 each (total 28) of *Candida diddensiae*,* Candida quercitrusa*,* Candida sake*,* Candida sojae*,* Candida sphaerica*, *Candida vitiphila*,* Candidozyma duobushaemuli (Candida duobushaemulonii)*, *Cystobasidium *sp*.*,* Diutina rugosa (Candida rugosa)*,* Hyphopichia burtonii*,* Issatchenkia terricola*,* Lachancea fermentati*,* Magnusiomyces clavatus (Geotrichum clavatum)*,* Meyerozyma neustonensis*,* Moesziomyces aphidis (Pseudozyma aphidis)*,* Naganishia alba*,* Naganishia globosa*,* Nakazawaea ishiwadae*, *Nakazawaea siamensis*,* Saccharomycopsis fibuligera*,* Staremella bacillaris*,* Starmerella stellata*,* Tardiomyces digboiensis* (*Candida digboiensis*),* Torulaspora delbrueckii*,* Trichosporon coremiiforme*,* Trichosporon ovoides*,* Wickerhamiella spandovensis*, and* Zygosaccharomyces hellenicus*.

### Clinical specimens and anatomical sites

In this study, blood culture isolates showed the greatest range of organisms with 60 separate species (Table 1). Sputum and oral isolates were the second and third most diverse, with 54 and 48 species, respectively. CNS isolates had the narrowest spectrum, with 11 species.

### Common species

*Candida albicans* and *Nakaseomyces glabratus* were the most frequently isolated organisms across all sample types analyzed, identified 7,381 and 4,279 times, respectively, comprising 41.86% and 24.27% of total samples. *Candida parapsilosis*, *Candida dubliniensis,* and *Candida tropicalis* were the third, fourth, and fifth more frequent species at 7.18% (*N* = 1,266), 5.12% (*N* = 903), and 4.83% (*N* = 851), respectively.

Reviewing by sample site (Table 3), *C. albicans* was the most common yeast from: genital-recurrent infection (79.80%, 1,529/1,916), genital infection not identified as recurrent infection (62.20%, 2,417/3,886), oral (42.20%, 825/1,955), pleural fluid (39.62%, 42/106), speech box (27.70%, 41/148), and sputum (24.57%, 501/2,039) isolates; *N. glabratus* was the most frequent in urine (44.51%, 802/1,802), blood (34.66%, 1,573/4,538), and BAL fluid (22.46%, 155/690) samples; *C. parapsilosis* was the most frequent yeast in eye samples, including contact lenses (39.37%, 100/254) and CF sputum specimens (19.23%, 30/156); while *Cryptococcus neoformans* was the most common organism in samples from the CNS (77.30%, 109/141).

**TABLE 3:** Frequency of species found in each sample site^*a*^^,^^*b*^

Organism	Frequency												
	Overall	Blood	CNS	Urine	Genital	Genital recurrent	Eyes	Speech box	Pleural fluid	BAL fluid	Sputum	CF sputum	Oral
*Candida albicans*	41.86	29.99	7.09	24.25	**62.20**	**79.80**	22.83	**27.70**	**39.62**	20.14	**24.57**	13.46	**42.20**
*Nakaseomyces glabratus*	24.27	**34.66**	2.13	**44.51**	20.48	9.81	0.79	13.51	16.98	**22.46**	15.55	6.41	20.20
*Candida parapsilosis*	7.18	11.39	6.38	6.88	4.25	2.45	**39.37**	8.78	7.55	5.80	7.80	**19.23**	2.76
*Candida dubliniensis*	5.12	3.28	2.13	1.44	1.65	1.77	1.18	0.68	6.60	13.33	15.40	11.54	9.82
*Candida tropicalis*	4.83	4.45	0.71	6.60	1.08	0.26	1.18	25.68	5.66	11.16	11.03	1.92	6.65
*Saccharomyces cerevisiae*	2.72	0.44	–^*c*^	0.72	3.09	1.77	–	1.35	7.55	7.23	7.36	–	4.55
*Clavispora lusitaniae*	2.56	2.58	0.71	4.27	1.67	1.44	1.18	2.03	3.77	3.91	3.68	12.82	1.64
*Pichia kudriavzevii*	2.50	2.14	–	3.94	2.14	0.78	–	13.51	3.77	2.32	2.50	0.64	4.19
*Meyerozyma guilliermondii*	1.37	1.15	0.71	0.89	1.03	0.63	13.39	1.35	0.94	1.74	1.47	–	2.15
*Cryptococcus neoformans*	1.16	1.81	**77.30**	–	–	–	–	–	0.94	1.01	0.15	1.28	–
*Rhodotorula mucilaginosa*	0.63	1.37	–	0.11	0.51	0.57	3.54	–	0.94	0.46	0.05	–	0.15
*Kluyveromces marxianus*	0.61	0.79	–	0.83	0.23	0.16	–	0.68	–	1.88	1.13	–	0.41
*Meyerozyma caribbica*	0.57	0.42	–	0.28	0.15	–	–	0.68	1.89	1.74	1.52	1.28	1.18
*Candidozyma auris*	0.50	0.77	0.71	2.11	0.03	–	–	–	–	0.29	0.44	0.64	0.10
*Candida metapsilosis*	0.41	0.64	–	0.33	0.26	0.16	0.39	0.68	–	0.87	0.64	–	0.15
*Magnusiomyces capitatus*	0.36	0.71	–	0.22	–	–	–	–	–	0.58	0.88	0.64	0.20
*Candida orthopsilosis*	0.24	0.18	–	0.33	0.08	0.10	1.57	–	–	0.29	0.69	–	0.20
*Cyberlindnera fabianii*	0.22	0.20	–	0.44	0.13	0.05	–	–	–	0.43	0.44	0.64	0.10
*Geotrichum candidum*	0.19	–	–	–	–	–	–	0.68	–	0.43	1.03	2.56	0.20
*Naganishia diffluens*	0.19	–	1.42	–	0.08	0.05	3.54	–	–	1.45	0.15	1.92	0.10
*Trichosporon asahii*	0.19	0.20	–	0.61	–	–	0.39	–	–	0.29	0.44	0.64	–
*Wickerhamomyces anomalus*	0.19	0.26	–	–	0.08	–	1.97	–	–	0.14	0.39	–	0.26
*Pichia cactophila*	0.14	0.11	–	0.22	0.10	0.05	–	–	0.94	0.43	0.05	–	0.31
*Lloderomyces elongisporus*	0.12	0.20	–	–	0.08	0.05	–	–	–	–	0.15	1.28	0.15
*Nakaseomyces nivariensis*	0.11	0.18	–	–	0.13	0.05	0.39	–	–	0.14	0.05	–	0.15
*Yarrowia lipolytica*	0.11	0.13	–	–	0.03	–	2.36	–	0.94	0.29	–	–	0.20

^*a*^ Bold indicates most frequent species for that sample site.

^*b*^ CNS, central nervous system including cerebrospinal fluid; BAL, bronchoalveolar lavage; CF, cystic fibrosis.

^*c*^ –, not applicable.

### Less common and rare species

*Clavispora lusitaniae* (formerly *Candida lusitaniae*) was isolated on 451 occasions (2.56%, 451/17,631) and was represented in all sample types. The sample type with the highest frequency of *Cl. lusitaniae* detection was CF sputum, where it accounts for 12.82% of isolates (Table 3). *Candidozyma* (ex-*Candida*) *auris* was isolated on 89 occasions (0.50%, 89/17,631), and urine samples had the highest frequency of *C. auris* detection at 2.11%.

Regarding basidiomycetes, the most frequently identified was *Cryptococcus neoformans* at 1.16% (204/17,631) of all isolates, followed by *Rhodotorula* spp. at 0.71% (125/17,631), of which *R. mucilaginosa* predominated (*N* = 112). *Trichosporon* spp. were identified on 51 occasions (0.29%, 51/17,631), with *T. asahii* being the most common (*N* = 33). *Malassezia* spp. were identified on 28 occasions, with *Malassezia furfur* (*N* = 15) and *Malassezia pachydermatis* (*N* = 13), mainly found in blood culture samples, accounting for 60.0% (9/15) and 69.2% (9/13) of isolations, respectively.

Of the rarer organisms, 69 species were isolated on fewer than 20 occasions each, with 28 that were only present in a single sample (Table 4). A few of these rare organisms demonstrated noteworthy site preponderance: *Apiotrichum mycotoxinivorans* was identified 17 times and exclusively from CF sputum samples, an association that has previously been reported (27) with evidence of clinical significance in this patient cohort; the unicellular microalgae *Prototheca* spp. were identified 11 times and solely from blood isolates, again in keeping with historical reports highlighting their capacity to cause invasive/disseminated disease in humans and other animals (28); *Hanseniospora guilliermondii* was identified on seven occasions and only from sputum or oral samples.

**TABLE 4:** Distribution of sample sites for the less frequent organisms^*a*^

Organism	Isolates												
	Overall	Blood	CNS	Urine	Genital	Genital recurrent	Eyes	Speech box	Pleural fluid	BAL fluid	Sputum	CF sputum	Oral
*Apiotrichum mycotoxinivorans*	17	–^*b*^	–	–	–	–	–	–	–	–	–	17	–
*Wickerhamiella pararugosa*	16	–	–	2	2	–	–	1	–	1	4	–	6
*Malassezia furfur*	15	9	–	4	1	–	–	–	1	–	–	–	–
*Malassezia pachydermatis*	13	9	–	2	2	–	–	–	–	–	–	–	–
*Rhodotorula dairenensis*	13	7	–	1	1	–	1	–	–	–	1	–	2
*Candidozyma haemuli*	12	4	–	–	–	–	6	–	1	1	–	–	–
*Tardiomyces blankii*	12	2	–	–	1	–	–	–	–	1	3	4	1
*Trichosporon inkin*	12	7	–	–	3	–	1	–	–	–	–	1	–
*Prototheca* spp.	11	11	–	–	–	–	–	–	–	–	–	–	–
*Pichia kluyveri*	10	1	–	2	2	–	–	–	–	–	5	–	–
*Pichia fermentans*	9	1	–	–	–	–	–	–	–	1	3	–	4
*Pichia barkerii*	8	–	–	2	–	–	–	1	–	–	–	–	5
*Wickerhamomyces onychis*	8	2	–	–	1	–	–	–	–	3	–	1	1
*Hanseniospora guilliermondii*	7	–	–	–	–	–	–	–	–	–	2	–	5
*Pichia manshurica*	7	1	–	–	–	–	–	1	–	1	3	–	1
*Candida palmioleophila*	6	1	–	–	2	–	1	–	–	1	1	–	–
*Debaryomyces hansenii*	6	–	–	–	–	–	3	–	–	–	1	1	1
*Kodamaea ohmeri*	6	3	–	–	–	–	–	–	–	–	2	–	1
*Cyberlindnera jadinii*	5	1	–	–	1	–	–	–	–	2	1	–	–
*Metschnikowia pulcherrima*	5	–	–	–	–	–	–	–	–	1	3	1	–
*Pichia norvegensis*	5	2	–	1	1	–	–	–	–	–	–	–	1
*Sporopachydermia cereana*	5	3	–	–	–	–	–	–	–	–	1	–	1
*Sungouiella intermedia*	4	–	–	–	1	–	–	–	–	1	2	–	–
*Starmerella magnoliae*	4	3	–	–	–	–	–	–	–	–	–	–	1
*Starmerella sorbosivorans*	4	2	–	–	–	–	–	–	–	–	1	–	1
*Trichosporon japonicum*	4	1	–	1	–	–	1	–	–	1	–	–	–
*Blastobotrys adeninivorans*	3	–	–	–	–	–	–	–	–	–	–	3	–
*Candida zeylanoides*	3	1	–	–	–	–	–	–	–	–	–	1	1
*Cutaneotrichosporon cutaneum*	3	1	–	–	–	–	2	–	–	–	–	–	–
*Cutaneotrichosporon mucoides*	3	2	–	1	–	–	–	–	–	–	–	–	–
*Diutina catenulata*	3	2	–	1	–	–	–	–	–	–	–	–	–
*Geotrichum silvicola*	3	–	–	–	–	–	–	–	–	–	2	1	–
*Kazachstania telluris*	3	1	–	–	–	1	–	–	–	–	–	–	1
*Pichia occidentalis*	3	–	–	–	–	–	–	1	–	–	–	2	–
*Athrographis kalrae*	2	–	–	–	–	–	–	–	–	–	–	2	–
*Pichia ethanolica*	2	–	–	1	1	–	–	–	–	–	–	–	–
*Candida sanyaensis*	2	–	–	–	1	–	–	–	–	–	–	–	1
*Hansensiospora uvarum*	2	–	–	–	–	–	–	–	–	–	1	–	1
*Millerozyma farinosa*	2	–	–	–	–	–	–	–	–	–	1	1	–
*Nakaseomyces bracarensis*	2	1	–	–	–	–	–	–	–	–	1	–	–
*Starmerella stellata*	2	–	–	–	–	–	–	–	–	–	1	1	–

^*a*^ CNS, central nervous system including cerebrospinal fluid; BAL, bronchoalveolar lavage; CF, cystic fibrosis. Plus 1 each (isolates from blood or CSF in bold) of *Candida diddensiae*, ***Candida quercitrusa***,* Candida sake*,* Candida sojae*,* Candida sphaerica*, *Candida vitiphila*, ***Candidozyma duobushaemulonii***, ***Cystobasidium*** sp***.*****,** ***Diutina rugosa***, ***Hyphopichia burtonii***,* Issatchenkia terricola*,* Lachancea fermentati*, ***Magnusiomyces clavatus*****,***** Meyerozyma neustonensis***, ***Moesziomyces aphidis***,* Naganishia alba*,* Naganishia globosa*,* Nakazawaea ishiwadae*, *Nakazawaea siamensis*,* Saccharomycopsis fibuligera*,* Staremella bacillaris*,* Starmerella stellata*, ***Tardiomyces digboiensis***,* Torulaspora delbrueckii*,* Trichosporon coremiiforme*,* Trichosporon ovoides*,* Wickerhamiella spandovensis*, and ***Zygosaccharomyces hellenicus**.*

^*b*^ –, not applicable.

### Fluconazole resistance

Fluconazole susceptibility testing was performed on 3,169 isolates: *C. albicans* (*N* = 1,106), *C. parapsilosis* (*N* = 424), *C. tropicalis* (*N* = 278), and *N. glabratus* (*N* = 1,361; Table 5). Considerable resistance was particularly present in isolates identified from superficial samples (i.e., genital and oral cavity). For *C. albicans* recovered from oral sites, 35.0% (49/140) were non-susceptible to fluconazole (MIC ≥ 4 mg/L); among isolates recovered from genital sites, this figure was 19.2% (91/475), increasing to 21.0% (44/210) when indicated as recurrent infection. These fluconazole resistance rates for *C. albicans* isolates from vaginal infections are remarkably similar to those reported recently over a period of 10 years in a single referral center in the US (overall resistance rate = 22.8%) (29). In contrast, all the 190 *C. albicans* isolates from blood culture were susceptible to fluconazole (MIC < 4 mg/L). There was less fluconazole resistance seen in *C. parapsillosis* and *C. tropicalis* from superficial sites; for example, oral samples showed 3.7% (1/27) and 1.7% (1/58) non-susceptibility, respectively. As expected, isolates of *N. glabratus* displayed higher MICs to fluconazole; this organism showed a similar pattern to *C. albicans*, with more resistance found in isolates from superficial sites compared to deep sites. *N. glabratus* isolates from genital samples (combined recurrent and non-recurrent) had 22.6% (81/359) non-susceptibility rates (MIC ≥ 32 mg/L), falling to 7.8% (49/626) in blood culture isolates.

**TABLE 5:** Fluconazole antifungal susceptibility testing results^*a*^^,^^*c*^

Organism	*N*	Site	Fluconazole MIC (mg/L)											
			≤0.125	0.25	0.5	1	2	4	8	16	32	≥64	%NS	%R
*C. albicans*	190	Blood culture	10	144	25	6	5	–^*b*^	–	–	–	–	0.0	0.0
	91	Respiratory	3	63	10	5	5	3	2	–	–	–	5.5	2.2
	140	Oral	5	69	10	3	4	9	10	10	10	10	35.0	28.6
	475	Genital	11	263	30	36	44	46	34	4	–	7	19.2	9.5
	210	Genital recurrent	4	118	11	12	21	17	23	2	–	2	21.0	12.9
														
*C. parapsilosis*	240	Blood culture	2	53	120	57	5	1	–	2	–	–	1.3	0.8
	80	Respiratory	–	18	28	28	1	2	–	1	–	2	6.3	3.8
	27	Oral	–	6	13	4	3	–	1	–	–	–	3.7	3.7
	50	Genital	–	9	23	12	4	–	2	–	–	–	4.0	4.0
	27	Genital recurrent	–	4	9	5	7	1	1	–	–	–	7.4	3.7
														
*C. tropicalis*	86	Blood culture	–	22	26	26	9	–	–	3	–	–	3.5	3.5
	123	Respiratory	1	30	48	26	9	–	6	1	2	–	7.3	7.3
	58	Oral	–	21	18	14	4	–	–	1	–	–	1.7	1.7
	11	Genital	–	3	4	3	–	1	–	–	–	–	9.1	0.0
														
*N. glabratus*	626	Blood culture	–	–	2	24	128	250	137	36	19	30	NA	4.8
	236	Respiratory	–	2	2	11	42	107	44	18	8	2	NA	0.9
	140	Oral	–	–	3	10	24	66	28	6	2	1	NA	0.7
	243	Genital	–	1	–	7	35	69	45	34	21	31	NA	12.8
	116	Genital recurrent	–	1	1	3	17	35	20	10	16	13	NA	11.2

^*a*^ CLSI species-specific breakpoints indicated by shading as per CLSI, where MICs shaded in gray indicate susceptible dose-dependent (SDD) or the top end of SDD for *N. glabratus*. *N*, number of samples; MIC, mean inhibitory concentration; %NS, % non-susceptible; %R, % resistant; NA, not applicable as for *N. glabratus*. All MICs <32 are considered SDD.

^*b*^ –, not applicable.

^*c*^ Gray shaded MIC values indicate susceptible dose-dependent except for *N. glabratus*, where all MIC values <32 mg/L are susceptible dose-dependent.

## DISCUSSION

We identified 95 different species in total, comprising 94 species of yeast and one genus of microalgae. *Candida albicans* and *Nakaseomyces glabratus* were by far the most common, consistent with our previously published data as well as most international epidemiological surveys (12, 30, 31). The prevalence seen at the MRL has remained stable over time; here, *C. albicans* accounts for 41.86% of all yeast isolates and *N. glabratus* for 24.27%, with our previous data (2002–2016) showing *C. albicans* at 39.3%–49.3% and *N. glabratus* at 20.5%–22.6% (12, 27). *C. parapsilosis* was the third most common, and these three organisms accounted for the most frequent isolates from every sample site, apart from the CNS, where *Cryptococcus neoformans* predominated. In keeping with its relative rarity in the UK, there were no isolations of *Cryptococcus gattii* in the study period. Interestingly, with caveats concerning the potential skewing of referrals to the MRL toward less common yeast species, *N. glabratus* was the most frequent species associated with bloodstream infections in the UK in the current study. This mirrors the situation recently reported in the United States, where cases of fungemia due to *N. glabratus* exceeded those due to *C. albicans* for the period 2016–2024 (32). Similar to the recently published global review of the repertoire of clinical yeasts (20), here *Candida dubliniensis* was highly represented, predominantly in superficial anatomical sites (oropharynx, respiratory secretions), but in agreement with recent US reports, it was a rarer cause of invasive disease/candidemia (32).

Sputum and oral samples showed a predictably wide range of different species, in keeping with the diversity of oral flora, as well as the many yeasts that can be introduced transiently to the oral cavity from food and beverages. To provide one small illustration, at least nine of the yeasts we identified among oral-related isolates can be found on grapes or are used in the winemaking industry: *Hanseniospora uvarum*, *Metschnikowia pulcherrima*, *Pichia kluyveri*, *Pichia fermentans*, *Pichia occidentalis*, *Pichia manshurica*, *Starmerella stellata*, *Torulaspora delbrueckii*, and *Wickerhamomyces anomalus* (33). It is quite likely that many of these isolates were referred to the MRL following failure to identify in the local laboratory, the utility of which is doubtful given the non-sterile environment of the oral cavity/upper respiratory tract and the questionable pathogenicity of these organisms (with the exception of species in the *Cryptococcus neoformans* and *Cr. gattii* complexes) at this site.

Less expected was the impressive range of organisms isolated and identified from blood cultures, representing the most diverse sample type with 60 different species. In part, this may relate to the high sample numbers received (*N* = 4538), but also the requirement for species-level identification from sterile sites (34) and hence referral of all organisms less readily identified in local laboratories. The MRL also performs formal identification of all isolates sent for susceptibility testing, which will include most blood culture isolates. The individual clinical relevance of each of these organisms is uncertain, and a proportion are likely to represent contamination with skin flora during sample collection; however, one should not rush to discount any yeast isolated in blood culture. Previous publications have similarly identified myriad yeasts in patient samples (20); however, correlation with clinical significance is often lacking. Humans will encounter a vast number of yeasts from the environment, pets, food, and drink, in addition to those that naturally colonize different anatomical sites. Isolation of such organisms from non-sterile sites may be entirely irrelevant depending on sample type, host factors, and clinical context.

There is a major limitation to our data, which we have commented on previously in similar reports. Isolates referred to the MRL will be naturally skewed toward the unusual, as (i) referring laboratories are less likely to be able to robustly identify them locally due to their absence or under-representation in commercially available MALDI-TOF mass spectra libraries, and (ii) many of the less common species have unusual or unexpected resistance patterns (12, 21). The advantage this bias provides is a wider oversight over rarely encountered organisms.

Reviewing our data on the increasingly common pathogen, *Candidozyma auris,* revealed a 2.11% prevalence (38/1,802) in urine isolates, a greater frequency than found in other sample types. This is a significant finding given the limited antifungal treatment options that provide good urinary penetration, especially after discounting fluconazole, to which the overwhelming majority of *C. auris* isolates have acquired resistance. Low prevalence of *C. auris* in samples obtained from normally sterile sites demonstrates that invasive infections caused by *C. auris* were infrequent in UK hospitals during this study period, in keeping with recent updated national data (35). In this respect, it should be noted that the data concerning *C. auris* prevalence in the current report are incomplete: the vast majority of *C. auris* isolates referred to the MRL from across the UK continue to be from screening swabs of superficial skin sites (axilla, groin, and nares) to detect patient colonization and the LIMS searches performed to capture the data presented here did not include such sample types.

The triazole drug fluconazole continues to be one of the most commonly prescribed antifungal agents, both for prophylaxis in high-risk patients and as a treatment option for invasive infections caused by *Candida* and allied genera. While there is growing concern regarding antifungal resistance globally, we show that for *C. albicans*, the resistance to fluconazole is primarily restricted to superficial isolates in our tested collection, whereas those causing invasive infections retain susceptibility to this agent. None of the 190 invasive *C. albicans* isolates found in blood cultures was resistant to fluconazole in this study. In contrast, around 20% of *C. albicans* isolates from genital samples were non-susceptible, likely resulting from prolonged and/or repeated antifungal exposures of isolates from superficial sites, possibly exacerbated by the ready availability of over-the-counter oral and topical azole preparations. A similar picture was seen with *N. glabratus*, with a shift toward higher MICs seen in isolates from genital samples compared to those recovered from blood cultures. Although not desirable, antifungal resistance in yeasts causing superficial site infection is of lower clinical significance, as some resistance mechanisms may be overcome by the higher local concentration of antifungals possible with topical application. In this respect, it is also reassuring that fluconazole resistance rates in UK isolates of *C. parapsilosis* from deep sites were extremely low (<1%), indicating that the clonal spread of fluconazole-resistant isolates reported in parts of the USA, South America, and mainland Europe (36, 37) has not so far extended to the UK to a significant degree. Similarly, the recently reported increase in fluconazole resistance in isolates of *C. tropicalis* from cases of candidemia, where resistance rates exceeded 18% in a US nationwide survey (37), does not currently appear to be replicated in UK isolates.

### Conclusions

A wide variety of yeast species can be encountered in human clinical samples; however, these are of variable relevance. Each result should be individually corroborated with sample type, host factors, and clinical context to ascertain significance. Significant fluconazole resistance in common *Candida* spp. and allied genera is, fortunately, currently more limited to superficial rather than invasive isolates in the United Kingdom.
